# Fahr Syndrome Presenting With Status Epilepticus After COVID-19 Infection

**DOI:** 10.1210/jcemcr/luad072

**Published:** 2023-06-28

**Authors:** Helen Yifter Bitew, Immaculate Kambutse, Aloys Tuyizere, Gakumba Claude

**Affiliations:** College of Medicine and Health Sciences, University of Rwanda, Kigali, Rwanda; Department of Internal Medicine, King Faisal Hospital, Kigali, Rwanda; College of Health Sciences, Addis Ababa University, Addis Ababa, Ethiopia; College of Medicine and Health Sciences, University of Rwanda, Kigali, Rwanda; Department of Internal Medicine, King Faisal Hospital, Kigali, Rwanda; College of Medicine and Health Sciences, University of Rwanda, Kigali, Rwanda; Department of Internal Medicine, King Faisal Hospital, Kigali, Rwanda; College of Medicine and Health Sciences, University of Rwanda, Kigali, Rwanda; Department of Perioperative and Critical Care Services, King Faisal Hospital, Kigali, Rwanda

**Keywords:** Fahr syndrome, hypoparathyroidism, COVID 19 infection

## Abstract

Hypoparathyroidism is a rare metabolic disease. In addition to symptoms of hypocalcemia, chronic hypoparathyroidism can result in brain calcifications leading to Fahr syndrome. Hypoparathyroidism most commonly results as a postsurgical complication, with autoimmune disease the second most common etiology. Here we report a 48-year-old man with symptoms of chronic hypocalcemia who presented with status epilepticus following severe coronavirus disease 2019 (COVID-19) infection. In addition to severe hypocalcemia, he was found to have an inappropriately low serum parathyroid hormone level and basal ganglia calcifications visualized on head computed tomography scan. He was treated with intravenous calcium infusion prior to transition to orally administered calcium, calcitriol, and high-dose cholecalciferol (vitamin D3).

## Introduction

Fahr syndrome is a rare degenerative neuropsychiatric disorder characterized by seizures and extrapyramidal and neuropsychiatric symptoms. Fahr syndrome results from bilateral calcifications of the basal ganglia, thalamus, and other parts of the brain. It has a prevalence of less than 1/1 000 000 [1]. Fahr syndrome most commonly results from chronic hypoparathyroidism but can also be attributed to other etiologies, including infectious, metabolic, and genetic disorders.

Hypocalcemia in primary hypoparathyroidism results from inadequate parathyroid hormone (PTH) secretion, which is associated with impaired calcium mobilization from bone, impaired calcium reabsorption from the distal nephron, and impaired conversion of PTH-stimulated 1α-hydroxylase activity, the enzyme which mediates conversion of 25-dihydroxyvitamin D to 1,25-dihydroxyvitamin D within the kidney, leading to impaired intestinal calcium absorption.

Several studies have reported a higher prevalence of hypocalcemia in hospitalized patients with COVID-19 infection, with some having shown hypocalcemia to be an independent risk factor that predicts hospitalization, intensive care unit (ICU) admission and mortality [2-4]. Di Filippo et. al., compared the prevalence of hypocalcemia in hospitalized patients either with or without COVID-19, finding a higher prevalence of hypocalcemia in those infected with COVID-19 [5].

Herein, we report a 48-year-old man who presented with seizures associated with COVID-19 infection. Subsequent evaluation demonstrated concomitant hypocalcemia, hyperphosphatemia, inappropriately low PTH level, low 25-hydroxyvitamin D level, and bilateral brain calcifications on head computed tomography (CT) scan.

## Case Presentation

A 48-year-old man was admitted on transfer to King Faisal Hospital Rwanda from a district hospital after he had presented with repetitive tonic-clonic seizures. He had visited a private clinic one day prior to presentation to the district hospital due to symptoms which included confusion, fatigue, and difficulty speaking. He was found to be positive for COVID-19 and was sent home with favipiravir treatment.

His family reported that the patient had an altered gait, which had been present and progressive over the preceding 5 years. This was described as wide-based, slow, and progressively hunched. He also had a history of paresthesias and numbness around the mouth, fingers, and toes which had been present for many years. Review of his medical records showed presentation with palpitations and fatigue 8 years previously, and shortness of breath 4 years previously. Two years before the current presentation, he had visited a psychologist for forgetfulness and inattention—symptoms that led to job loss. Laboratory records showed a serum calcium concentration of 1.3 mmol/L (5.2 mg/dL) [reference range of 2.15-2.5 mmol/L; 8.6-10 mg/dL] 8 years before the current presentation. He had no history of trauma, hypertension, diabetes, cerebral vascular accident, thyroid disease, or autoimmune disease. He had no history of neck surgery or prior neck irradiation. He had no family history of epilepsy. He was divorced and lived with his family, drank alcohol occasionally, and did not smoke.

In the emergency department, the patient was unconscious and in respiratory distress. Vital signs were as follows: blood pressure = 143/80 mmHg; heart rate = 97 beats per minute; blood oxygen saturation = 93% on 10 liters of oxygen via nasal cannula; respiratory rate = 18 breaths/minute.

He had no pallor, jaundice, palpable peripheral lymph nodes, cyanosis, or clubbing. His nutrition status was good. He had bilateral diffuse fine crackles on both lung fields. His extremities were warm, he appeared to have good blood volume with regular pulses, and his heart sounds were without added sounds or murmurs. He had moderate central obesity and a soft, nontender abdomen with no palpable masses and normal bowel sounds. He had no signs of Albright hereditary osteodystrophy. His Glasgow coma scale was 11/15 (E4, V3, M4), pupils were equally reactive to light, and there was no meningismus or focal neurological deficits. Tonic-clonic rhythmic movement of the upper and lower extremities was observed during examination. He had spontaneous facial twitching and Trousseau sign was positive.

## Diagnostic Assessment

Initial laboratory investigation showed the following: serum total calcium concentration (0.65 mmol/L; 2.6 mg/dL [reference range of 2.15-2.5 mmol/L; 8.6-10 mg/dL]), serum phosphorus concentration (3.63 mmol/L; 11.2 mg/dL [reference range, 0.81-1.45 mmol/L; 2.5-4.5 mg/dL]), serum PTH concentration (1.3 pmol/L; 1.3-6.9 pmol/L), serum 25-hydroxyvitamin D concentration (7 ng/mL; 17.5 nmol/L [reference range, <10 ng/mL (<25 nmol/L) = severe deficiency]). Clinically, he was found to have sepsis from a pulmonary focus with associated acute kidney injury and liver dysfunction.

**Figure 1.**
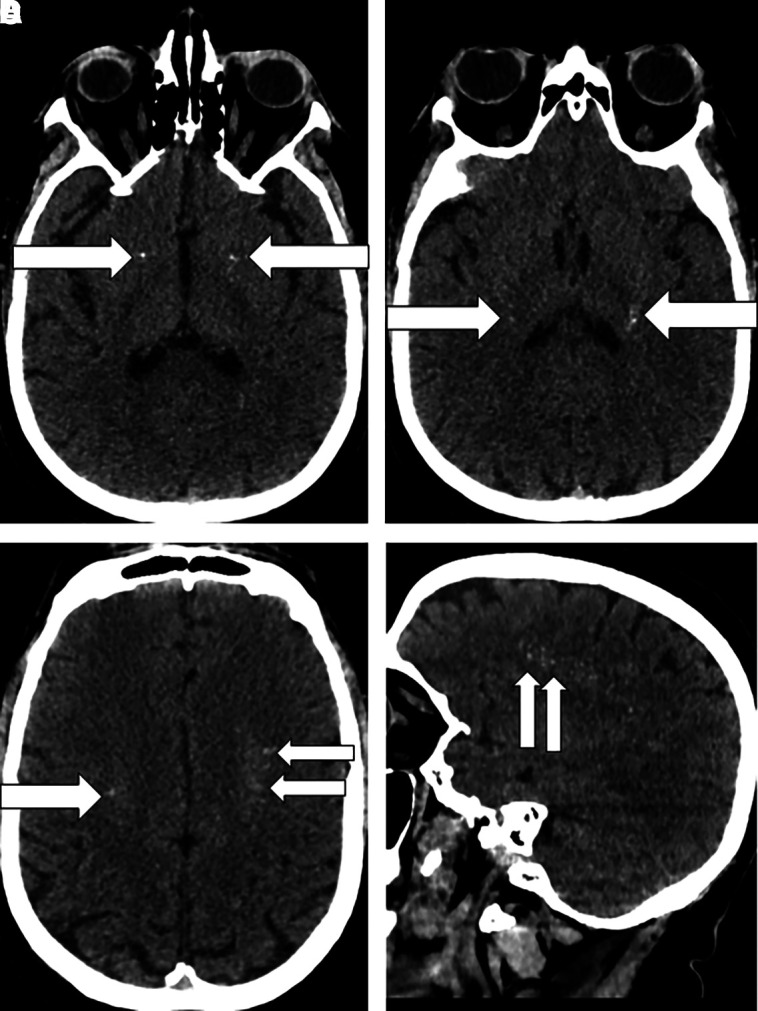
Brain CT scan showed bilateral asymmetrical punctate calcifications in the basal ganglia. Panel A, axial image with putamen calcifications (arrows). Panel B, axial image with calcifications in the posterior limb of the internal capsule (arrows). Panel C, axial image with bilateral and asymmetrical punctate and linear calcifications in the bilateral corona radiata (arrows). Panel D, sagittal image with punctate and linear calcifications in the left corona radiata (arrows).

Brain CT imaging demonstrated dense calcifications within the bilateral white matter (Fig. 1). Chest radiograph showed bilateral opacities. Abdominal ultrasound was normal.

## Treatment

The patient was diagnosed with primary hypoparathyroidism and severe hypovitaminosis D in the clinical setting of severe COVID-19 infection complicated by pneumonia and sepsis with associated multiorgan dysfunction (acute kidney injury and liver dysfunction). The patient required mechanical ventilation, intravenous antibiotics, intravenous phenytoin for seizure control, and intravenous calcium infusion. He was hospitalized for 50 days.

## Outcome and Follow-up

Following his prolonged hospitalization, the patient ultimately improved. At the time of hospital discharge, he was treated with oral calcium carbonate 3 g daily (2 g in the morning and 1 g in the evening), vitamin D3 (60 000 units weekly for 6 weeks), and calcitriol 0.5 mcg twice daily. At his last follow-up visit (11 months after hospital discharge), serum calcium concentration was 2.34 mmol/L (9.36 mg/dL) and serum phosphorus concentration was 1.3 mmol/L (4.0 mg/dL). His most recent 25-hydroxyvitamin D level was 90 ng/mL (224.6 nmol/L). Although spontaneous twitching over the right side of the face was present, he had not experienced any seizures since hospital discharge.

## Discussion

The rate of development, severity, and duration of hypocalcemia determine the clinical presentation in patients with primary hypoparathyroidism. With acute hypocalcemia, patients typically present with tingling and perioral and/or extremity numbness, as well as more serious symptoms including respiratory distress due to bronchospasm, voice changes due to laryngospasm, cardiac arrythmia, syncope, and/or neuropsychiatric manifestations including irritability, fatigue, and seizures. With chronic hypocalcemia, the clinical presentation is more variable and ranges from the absence of symptoms to life-threatening symptoms. Although calcification most frequently affects the kidneys, other organs including joints, eyes, skin, vasculature, and brain may be affected. Basal ganglia calcifications can occur in chronic hypoparathyroidism, with a reported prevalence of 52% in a hypoparathyroidism cohort [6].

Hypocalcemia can be acquired or hereditary. Serum calcium concentrations reflect a complex interplay between PTH, vitamin D, and the calcium ion itself, in addition to physiologic effects reflecting intestinal absorption, renal calcium handing, and skeletal homeostasis. In adults, most cases of hypocalcemia that are unrelated to medications (ie, antiresorptive agents such as bisphosphonates or denosumab) are related to disordered PTH or vitamin D levels.

The mechanism underlying brain calcifications in hypoparathyroidism likely reflects prolonged hyperphosphatemia and an elevated calcium-phosphate product, resulting from hyperphosphatemia associated with hypoparathyroidism in conjunction with long-term treatment with activated vitamin D and calcium. High serum phosphorus levels may also activate the inorganic phosphate transporter Pit1 and result in the expression of osteogenic molecules in the caudate nucleus and gray matter as mechanisms explaining basal ganglia calcifications. [7]

Up to 75% of cases of hypoparathyroidism result as a complication of prior neck surgery; other causes include autoimmunity, genetic, or idiopathic. Our patient has no history of surgery or irradiation. Due to limited resources, we were unable to evaluate autoimmunity markers or germline genetic testing. Our patient did not have type 1 diabetes, adrenal insufficiency, hypothyroidism, or candidiasis as part of a polyglandular autoimmune syndrome. Likewise, although both hypermagnesemia and hypomagnesemia can inhibit PTH secretion and function, our patient had a normal serum magnesium level.

 Our patient had previously sought care for palpitations, fatigue, and neuropsychiatric symptoms, all of which may have been attributable to chronic hypocalcemia, prior to presentation with status epilepticus precipitated by COVID-19 infection. There have been a few reports of decompensated hypoparathyroidism in the setting of COVID-19 infection [8-10], with patients with concomitant COVID-19 infection and hypocalcemia having higher rates of hospitalization and poor outcomes [3, 4]. There are recommendations to obtain blood calcium levels in patients with severe COVID-19 infection [5].

## Learning Points

- Unrecognized or poorly treated hypocalcemia can result in endocrine emergencies leading to significant morbidity or death.
- Patients presenting with symptoms and signs of hypocalcemia should be evaluated and treated promptly.
- In patients presenting with hypocalcemic emergency, timely diagnosis and treatment can be lifesaving.
- Expert recommendations suggest measuring serum calcium concentrations in all patients requiring hospitalization for COVID-19 infection.

## Data Availability

Data sharing is not applicable to this article as no datasets were generated or analyzed during the current study.

## Abbreviations

CT: computed tomography
PTH: parathyroid hormone
